# A review of consecutive cardiac arrests in 2002 and 2007 at a regional hospital in Denmark: a retrospective cohort study

**DOI:** 10.1186/1757-7241-20-S2-P2

**Published:** 2012-04-16

**Authors:** Henriette Ullerup-Aagaard, Søren Nielsen, Mikkel Brabrand

**Affiliations:** 1 Department of Cardiology, Odense University Hospital, Denmark; 2 Department of Anaesthesia, Odense University Hospital, Denmark; 3 Department of Medicine, Sydvestjysk Sygehus Esbjerg, Denmark

## Background

Most of the available literature on cardiac arrests is dealing with treatment. In this cohort study, we have sought to describe the events surrounding cardiac arrests such as time, cause, initial rhythm, and the final outcome.

## Methods

Retrospective analysis of all consecutive cardiac arrests at Sydvestjysk Sygehus Esbjerg in the years 2002 and 2007. The events were initially identified using a registry in the Department of Anaesthesia on all patient contacts. Using a unique personal identification number on all patients, we retrieved the charts and nurses notes and extracted the relevant data.

## Results

We included 267 cardiac arrests, 175 out-of-hospital and 92 in-hospital arrests. The cardiac arrests were distributed with 21 % in daytime (08-16), 27 % in the evening (16-00) and 24 % at night (00-08). The causes of cardiac arrests were acute coronary syndrome in 24 %, respiratory insufficiency in 13 %, hypotension in 5 %, arrhythmias in 3 %, other causes in 15 % and unknown in 40 %. The initial rhythm was asystole in 58 %, pulseless electrical activity in 18 %, pulseless ventricular tachycardia/ventricular fibrillation in 15 %, rhythm with perfusion in 2% and unknown in 7 %. In three patients, the treatment was stopped because of a Do Not Attempt Resuscitation order in the chart. In 64 patients (20 %) the treatment was stopped due to return of spontaneous circulation and 30 (47 %) patients survived to discharge.

## Conclusion

Cardiac arrests at Sydvestjysk Sygehus Esbjerg occur around the clock with an even distribution during the day, evening and night. Most cardiac arrests are caused by acute coronary syndrome and respiratory failure. We most often witnessed asystole as the presenting rhythm. In-hospital arrests had a better chance of having return of spontaneous circulation. Even in patients with return of spontaneous circulation the final outcome is poor. There was no difference in the final outcome in 2002 and 2007.

